# N^6^-methyladenosine as a biological and clinical determinant in colorectal cancer: progression and future direction

**DOI:** 10.7150/thno.52366

**Published:** 2021-01-01

**Authors:** Jinming Li, Lei Liang, Yongzhi Yang, Xinxiang Li, Yanlei Ma

**Affiliations:** 1 Department of Colorectal Surgery, Fudan University Shanghai Cancer Center, Shanghai, China.; 2 Department of Oncology, Shanghai Medical College, Fudan University, Shanghai, China.

**Keywords:** colorectal cancer, m^6^A, RNA modification, cancer progression, cancer treatment

## Abstract

Colorectal cancer (CRC) is one of the most prevalent cancers and one of the leading causes of cancer death. Recent studies have provided evidence that N^6^-methyladenosine (m^6^A), the most abundant RNA modifications in eukaryote, performs many functions in RNA metabolism including translation, splicing, storage, trafficking and degradation. Aberrant regulation of m^6^A modification in mRNAs and noncoding RNAs found in CRC tissues is crucial for cancer formation, progression, invasion and metastasis. Further, m^6^A regulators and m^6^A-related RNAs may become promising biomarkers, prognosis predictors as well as therapeutic targets. Here, we review the biological and clinical roles of m^6^A modification in CRC, and discuss the potential of m^6^A in clinical translation.

## Introduction

Colorectal cancer (CRC) is the third most prevalent cancer and the fourth leading causes of cancer death, with a rapidly increasing mobidity and mortality in developing countries and a stabilizing or decreasing trend in developed countries where the CRC burden remains among the highest worldwide 1. The pathological mechanism of CRC development and progression includes chromosomal instability, microsatellite instability-high (MSI-H) and cytosine-phosphate-guanine (CpG) methylation, resulting in the mutations of oncogenes, tumor suppressor genes and genes related to mismatch repair 2. Based on the novel understanding and advanced techniques, great improvement has been made to diagnose and treat CRC, which largely increases the overall survival. However, the prognosis of patients with advanced CRC is grim 3. Therefore, the molecular mechanisms underlying CRC tumorigenesis and metastasis need to be further elucidated.

**Figure 1:**
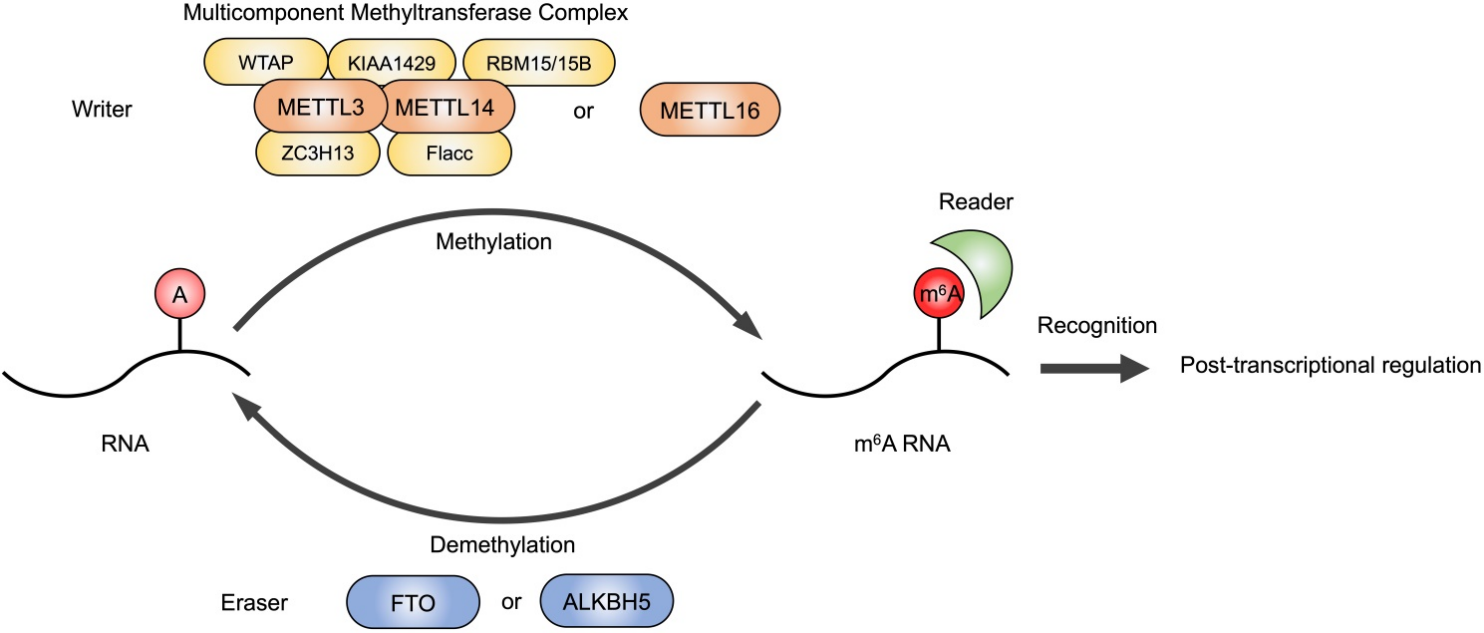
m^6^A modification on RNAs. The m^6^A modification is installed by writers, multicomponent methyltransferase complex (composed of METTL3, METTL14, WTAP, RBM15/15B, KIAA1429, ZC3H13 and Flacc) or METTL16 alone. FTO or ALKBH5 are m^6^A erasers that remove m^6^A modifications. Readers are required to recognize m^6^A and exert post-transcriptional regulation.

RNA modifications, including N^6^-methyladenosine (m^6^A), N^1^-methyladenosine (m^1^A), N^7^-methylguanidine (m^7^G), 5-methylcytidine (m^5^C), 2ʹ-O-methylation (Nm), pseudouridine (Ψ) and Inosine (I), are widely present in all types of RNAs, which mediate gene expression and participate in many biological processes, such as embryonic stem cell differentiation, circadian rhythms, temperature adaptation and meiotic progression 4. The m^6^A is the most abundant epigenetic modification in eukaryotes occurring in various types of RNA, including message RNA (mRNA), micro RNA (miRNA), long noncoding RNA (lncRNA) and circular RNA (circRNA) 5-8. As a reversible RNA methylation, m^6^A is installed by methyltransferase (also called writers), removed by demethylase (also called erasers), and recognized by some RNA-binding proteins (also called readers) (**Figure 1**). Typical consensus sequence of m^6^A modification sites is RRACH (R = G, A; H = A, C, U) 9,10. The m^6^A readers recognize RNA methylation and perform different biological functions, including RNA translation, splicing, storage, trafficking and degradation 11.

The m^6^A writer is a multicomponent methyltransferase complex in the nucleus, composed of a core protein heterodimer formed by methyltransferase like 3 (METTL3) and methyltransferase like 14 (METTL14), and other regulatory factors identified to interact with the METTL3/METTL14 complex to affect m^6^A deposition, including Wilms' tumor 1-associating protein (WTAP), Vir-like m^6^A methyltransferase-associated (KIAA1429/VIRMA), RNA-binding motif protein 15/15B (RBM15/15B), zinc finger CCCH domain-containing protein 13 (ZC3H13) and Fl(2)d-associated complex component (Flacc) 12-15. METTL16 is also a methyltransferase that can function alone and catalyze m^6^A of mRNAs, lncRNAs and U6 small nuclear RNA (U6 snRNA) 16. Fat mass and obesity-associated protein (FTO) and alkB homolog 5 (ALKBH5) are the erasers that have been identified to have demethylation activity 17,18. The dynamic regulation of m^6^A level in cellular RNAs is mediated by writers and erasers contributing to proper gene expression and protein production.

The well-studied m^6^A reader proteins are YT521-B homology (YTH) domain containing proteins, including YTHDC1-2 and YTHDF1-3, which bind RNA and recognize specific m^6^A sites to exert post-transcriptional function 19. For example, YTHDC1 participated in pre-mRNA processing and changed the length of 3'UTR, contributing to mRNA polyadenylation and splicing 20. YTHDF proteins altered translation efficiency and reduced stability of m^6^A modified RNAs 21. Insulin-like growth factor 2 mRNA-binding proteins (IGF2BPs) are also identified as m^6^A readers, which promote the stability and storage of their target mRNAs 22.

In addition to mRNA processing, m^6^A modifications regulate metabolism of noncoding RNAs, including miRNA, lncRNA and circRNA. The miRNA is a short regulatory RNA (∼22 nucleotides, nt) encoded in introns of coding and non-coding pre-mRNAs, which represses its target mRNA 23. Heterogeneous nuclear ribonucleoprotein A2B1 (HNRNPA2B1) is a reader protein that recruits the microprocessor complex to process primary miRNAs (pri-miRNAs) into mature miRNAs 24,25. The lncRNA is the RNA transcript generally longer than 200nt that does not encode protein, is associated to many cellular functions, one of which is m^6^A-dependent 26. lncRNA X-inactive specific transcript (XIST) mediated gene silence on the X chromosome via m^6^A installation and recognition 14. The circRNA is a type of single-stranded non-coding RNA that forms a covalently closed loop, participating in pathological processes through a m^6^A-dependent manner 27.

Owing to the m^6^A sequencing techniques that allow the detection of m^6^A with high efficiency, numerous RNAs and proteins are found to be a part of m^6^A regulation in CRC tumorigenesis. Methylated RNA immunoprecipitation sequencing (MeRIP-seq or m^6^A-seq) is used widely to detect m^6^A despite of the resolution of near 100 nt 9,10. New techniques, such as m^6^A individual-nucleotide-resolution cross-linking and immunoprecipitation (miCLIP) 28, m^6^A-sensitive RNA-endoribonuclease-facilitated sequencing (m^6^A-REF-seq or MAZTER-seq) 29,30, m^6^A-label-seq 31, deamination adjacent to RNA modification targets (DART-seq) 32, FTO-assisted m^6^A selective chemical labeling (m^6^A-SEAL) 33 are developed to map m^6^A at single-nucleotide resolution. Next generation sequencing methods on m^6^A epitranscriptome broaden our understanding of epigenomic marks in addition to traditional multi-omics analysis. Furthermore, the development of programmable RNA m^6^A editing by fusing CRISPR-Cas9 system with m^6^A writers or erasers without changing the primary sequence provides a powerful approach to uncover the mechanism under RNA modification during physiological and pathological processes 34.

It has been demonstrated that aberrant m^6^A deposition plays a critical role in various types of cancer 35. In this review, we summarize the dysregulation of m^6^A in CRC as well as the mechanisms how m^6^A regulators and m^6^A-related RNAs participated in CRC pathogenesis. We also discuss the clinical potential of targeting m^6^A in CRC in future.

## Aberrant Regulation of m^6^A in CRC

**Figure 2:**
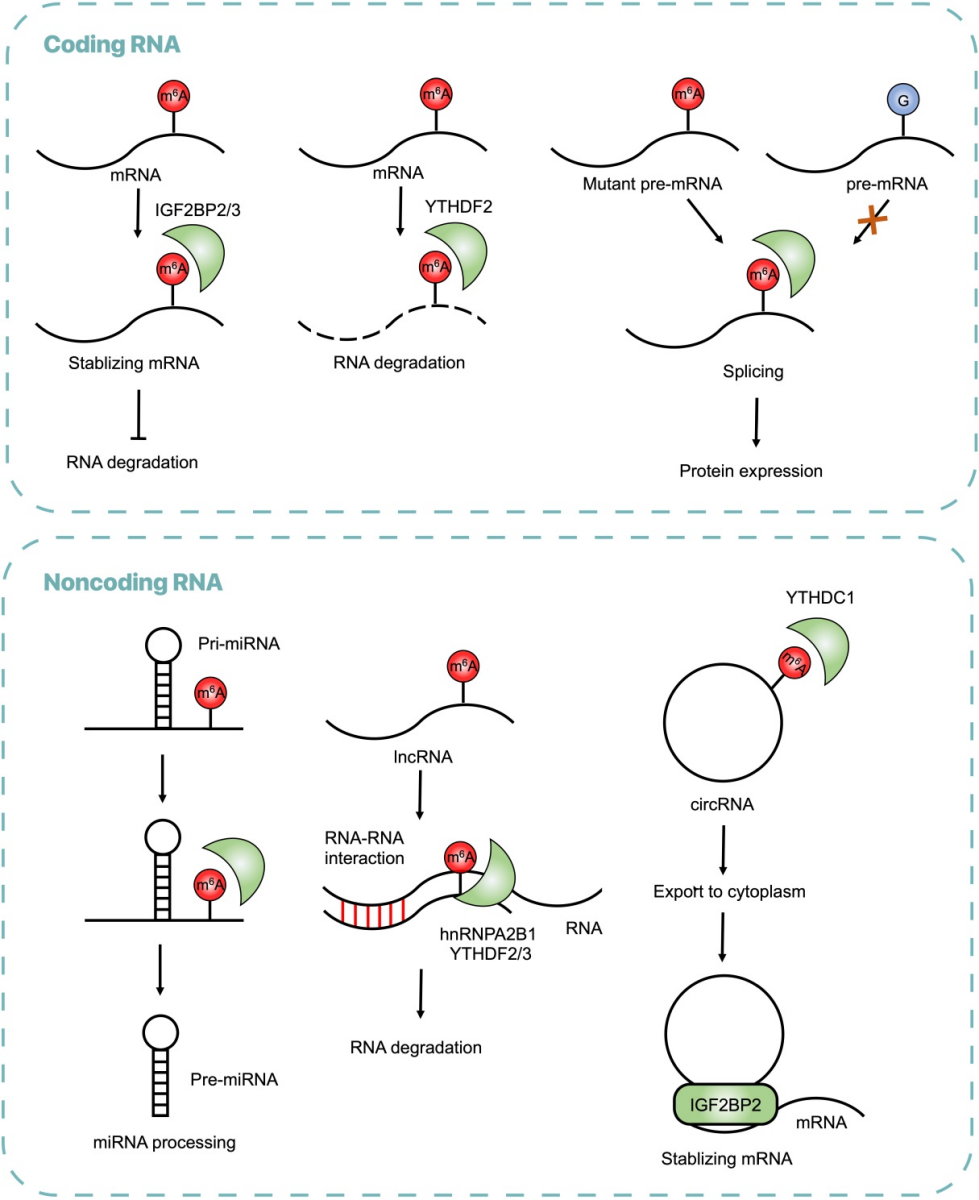
Regulatory Functions of m^6^A on RNAs in CRC. The m^6^A modifications recognized by reader proteins influence RNA metabolism, including RNA stabilization, splicing, processing, translocation and degradation.

Cumulative evidence has revealed that m^6^A modification widely alters gene expression in CRC. Global abundance of m^6^A and its regulators, including writers, erasers and readers are found dysregulated in CRC, which exerts oncogenic and/or antitumor function in CRC through targeting different types of RNA and various signal pathways (**Table 1**). The mechanisms are shown (**Figure 2**). Transcriptome-wide m^6^A methylome showed global m^6^A modifications in CRC and found 1343 dysregulated m^6^A peaks in mRNA compared with adjacent normal tissues, among which 625 were upregulated and 718 were downregulated, crucial in regulating glucose metabolism, RNA metabolism and cancer stem cells 36. In CRC, it was reported that m^6^A regulators, including METTL3, WTAP, FTO, YTHDF1, ZC3H13, HNRNPC, YTHDC, RBM15 and KIAA1429, were upregulated, while METTL14 and ALKBH5 were downregulated 37. Some downstream pathways of m^6^A modification were identified, such as phosphatidylinositol-3-kinase (PI3K)/Akt and mammalian target of rapamycin (mTOR) signal pathway 38.

### Dysregulation of m^6^A Writers

Aberrant expression of m^6^A writers in CRC leads to abnormal RNA metabolism, including mRNA stabilization, mRNA splicing, miRNA maturation and lncRNA degradation. Highly expressed METTL3 plays a critical role in CRC proliferation and progression by stabilizing target mRNAs through an m^6^A dependent manner. For instance, METTL3 raised m^6^A level of Cyclin E1 (CCNE1) mRNA in 3'UTR region and then increased CCNE1 mRNA stability in CRC cells 39. Overexpression of METTL3 was also found in metastatic CRC tissues, with its downstream target SRY(sex determining region Y)-box 2 (SOX2) methylated in coding sequence (CDS) regions and subsequently recognized by reader protein IGF2BP2, which prevented SOX2 mRNA degradation 40. Through interacting with the 5'/3'UTR regions of Hexokinase 2 (HK2) and the 3'UTR region of Glucose transporter 1 (GLUT1, also known as SLC2A1), METTL3 stabilized HK2 and GLUT1 transcripts to activate the glycolysis pathway and then promote CRC tumorigenesis 41.

In addition to the function of mRNA stabilization, the oncogenic role of METTL3 also affects noncoding RNA metabolism. Upregulated METTL3 markedly stabilized nascent lncRNA RP11 and increased its nuclear accumulation, which contributed to dissemination of CRC by escalating zinc finger E-Box binding homeobox 1 (Zeb1) 7. The miRNA is also the target of METTL3. METTL3 enhanced the metastatic potential of CRC by promoting the maturation of pri-miR1246 in a DGCR8-dependent manner. The miR1246 negatively regulated anti-oncogene Sprouty Related EVH1 Domain Containing 2 (SPRED2) which prevented cancer cell migration and invasion through Raf/MEK/ERK pathway 42.

Different from the oncogenic role of METTL3, METTL14 acts as an antitumor gene that suppresses CRC proliferation and metastasis. Loss of METTL14 correlated with unfavorable prognosis of CRC patients. METTL14 induced methylation of lncRNA XIST as well as mRNA SOX4 to downregulate their expression via YTHDF2-mediated RNA degradation 43,44 METTL14 also suppressed CRC cell growth via miR-375/YAP1 pathway as well as inhibited CRC cell migration and invasion via miR-375/SP1 pathway 45.

In contrary to the studies listed above that supported the oncogenic role of METTL3 in CRC, Ru et al. showed an antitumor role of METTL3, whose expression was associated with better survival and suppressed cancer growth and metastasis via p38/ERK pathways 46. Consistently, the controversial role of METTL3 has also been reported in other cancer types by different groups, such as glioblastoma 47,48. Like the dual role of METTL3, it was also reported that METTL14 acts as an oncogene in acute myeloid leukemia (AML) and breast cancer 49,50. The possible reason that may explain the controversial role of m^6^A writers is that the m^6^A sites and m^6^A modified RNAs reported in different studies are varied, which regulated different downstream targets and signal pathways, leading to the cancer heterogeneity. Hence, m^6^A and its regulators are promising biomarkers to distinguish cancer features.

It was reported that depletion of Mettl3 or Mettl14 in CRC cells enhanced the response of Patient-Derived Xenograft (PDX) mice to anti‐PD‐1 treatment by stabilizing the STAT1 and IRF1 mRNA and promoting IFN‐γ‐Stat1‐Irf1 signaling, which recruited cytotoxic tumor‐infiltrating CD8^+^ T cells in tumor microenvironment with escalated IFN‐γ, Cxcl9 and Cxcl10 51. In this case, METTL3 and METTL14 functioned synchronously to suppress anti-PD-1 treatment.

As a regulatory factor of m^6^A methyltransferase complex, the role of WTAP in CRC is far from understood. The association between WTAP expression and prognosis in patients with CRC varied from separate microarray databases 52,53, suggesting the multidimensional function of WTAP in tumor progression. Although some transcription factor binding sites have been identified in the promoter region of WTAP 54, the m^6^A related function of WTAP remains uncovered. Despite no direct evidence showing that WTAP could change m^6^A deposition in CRC cells, an interesting study demonstrated that the expression of WTAP was modulated by hypermethylation of CpG island, which might create a link between RNA modification and DNA modification. Transcriptional silence of CA4 in CRC tissues was mediated by hypermethylation with CpG sites within the CA4 promoter. CA4 interacted with WTAP and promoted its degradation through polyubiquitination and proteosome degradation, which in turn activated WT1 and increased the expression of downstream transcriptional factor Transducin β-Like Protein 1 (TBL1), resulting in the degradation of β-catenin and the inhibition of the Wnt signaling pathway 55. This finding indirectly connected m^6^A regulator WTAP with CpG methylation, indicating that RNA modification and DNA modification might regulate each other.

Other than m^6^A, m^5^C is a common RNA modification identified in transfer RNA (tRNA) 56, mRNA 57, vault RNA (vtRNA) 58 and also mitochondrial tRNA (mt-tRNA) 59. NOL1/NOP2/SUN domain family member 2 (NSUN2) is a RNA methyltransferase that introduces m^5^C into various RNAs to regulate RNA metabolism 56-59. The research group of the scientist Wengong Wang reported that the m^6^A level of miR-125b was positively associated with the expression of NSUN2, suggesting a role of NSUN2 in repressing miRNA via m^6^A 60. NSUN2, upregulated by proteinase-activated receptor 2 (PAR2) in CRC, was shown to interfere in the mature processing of miR-125b through a m^6^A dependent manner, thus regulating the expression of downstream gene Gab2, which contributed to CRC cell migration 61. It was puzzled that m^5^C methyltransferase NSUN2 could alter m^6^A level in RNA and exert post-transcriptional regulation since there was no evidence that NSUN2 could catalyze m^6^A. Follow-up study from Wengong Wang's group reported a cooperative function of m^6^A and m^5^C, which explained the role of NSUN2 via m^6^A. The m^5^C methylation mediated by NSUN2 facilitated the m^6^A methylation by METTL3/METTL14 in p21 mRNA, and reciprocally METTL3/METTL14-mediated m^6^A methylation enhanced NSUN2-mediated m^5^C methylation, implicating that joint m^6^A and m^5^C modification of the same RNA may influence each other and therefore coordinately affected protein expression, added a new layer of post-transcriptional regulation by RNA modification 62.

### Dysregulation of m^6^A Erasers

FTO has been considered essential for modulating fat mass and adipogenesis, whose single-nucleotide polymorphisms (SNPs) are related to the rising incidence of obesity and increasing risks of multiple cancer 63. FTO mRNA showed high mutation rate in MSI-H CRC, and corresponding frameshift peptides were produced 64. A number of studies presented different association between FTO SNPs and the risk of CRC based on different population and races 65-72. Recently, FTO has been identified as m^6^A and m^6^Am demethylase of mRNA and small nuclear RNA (snRNA) in the cell nucleus and cytoplasm 73-75, which performs m^6^A-related function in many types of cancer 76-79. FTO is also found upregulated in colorectal adenocarcinoma samples 34. Whether FTO participates in the CRC tumorigenesis via m^6^A requires further study.

ALKBH5 is the second identified m^6^A demethylase that modulates RNA metabolism 80. It was reported that ALKBH5 knockout (KO) in CRC cells enhanced efficacy of anti-PD-1 immunotherapy and improved mouse survival, indicating that ALKBH5 was a potential therapeutic target to improve immunotherapy outcome 81. During anti-PD-1 treatment, ALKBH5 decreased m^6^A near splice sites and modulated splicing of MCT4/SLC16A3 mRNA, which regulated lactate accumulation and infiltered immune cell in tumor microenvironment 81.

### Dysregulation of m^6^A Readers

The m^6^A readers are RNA binding proteins that recognize m^6^A modification at certain motif and control the modified RNA fate, so dysregulation of m^6^A readers may perturb RNA metabolism leading to pathological processes. For instance, upregulated YTHDC2 unwinded the 5′UTR of HIF-1α mRNA and promoted translation initiation, which contributed to colon cancer metastasis 82. In colonospheres, overexpressed YTHDF1 regulated stem cell-like activity, thus promoting tumorigenicity and cell cycle progression through Wnt/β-catenin pathway 83. In CRC, oncogenic transcription factor c-Myc may account for the amplified YTHDF1 84.

Apart from YTH domain family proteins, IGF2BP proteins were also identified as m^6^A readers 85. IGF2BP2 recognized m^6^A at CDS region of SOX2 mRNA maintaining its stability 40. An additional regulatory subunit of m^6^A reader was first identified and named as RNA-binding regulatory peptide (RBRP), which was a 71-amino acid peptide encoded by a previously annotated lncRNA LINC00266-1 86. Through binding to IGF2BP1, RBRP strengthened m^6^A recognition by IGF2BP1 on c-Myc mRNA to increase the mRNA stability and expression, thereby promoting tumorigenesis 86.

Reader proteins also regulate non-coding RNA, such as circRNA. CircNSUN2 is a circRNA derived from the exons 4 and 5 regions within the NSUN2 locus. It was clinically reported that upregulated expressions of circNSUN2 and HMGA2 mRNA are more prevalent in liver metastasis tissues than in primary CRC tissues 87. The cytoplasmic export of circNSUN2 was modulated by YTHDC1 at the GAACU motif. In the cytoplasm, circNSUN2 interacted with IGF2BP2 at the CAUCAU motif and then stabilized HMGA2 by forming a circNSUN2/IGF2BP2/HMGA2 RNA-protein ternary complex, which promoted liver metastasis 87. In this case, two reader proteins, YTHDC1 and IGF2BP2, recognized the same RNA at different motifs and exerted different functions respectively.

Dysregulation of m^6^A regulators causes abnormal m^6^A modifications in various RNA and aberrantly regulates the expression of RNA and their downstream pathways, which plays a critical role in cancer.

### Mutations in m^6^A sites in CRC

Generally, gene mutations are commonly found in cancer samples. However, little is known about the role of mutations in m^6^A sites in cancer. Mutated m^6^A sites of RNA may alter m^6^A deposition, which triggers aberrant post-transcriptional regulation and therefore leads to carcinogenesis. It was reported that he germline missense rs8100241 variant, located in the exon of Ankyrin Repeat and LEM Domain Containing 1 (ANKLE1) with a G>A change (Ala>Thr), was associated with decreased risk of CRC 88. Less microsatellites was found in the ANKLE1 [A] than the ANKLE [G] allele, suggesting the ANKLE [A] could function as a potential tumor suppressor that inhibited cancer cell proliferation by maintaining genomic stability. Variant ANKLE1 [A] was methylated by METTL3 while ANKLE [G] could not be methylated, which facilitatd the stability of ANKLE1 mRNA via m^6^A and promoted the expression of ANKLE1 protein, resulting in the reduced risk of CRC 88.

Generally, p53 is the most frequently mutated gene in cancer 89. In response to DNA damage stress and other oncogenic stresses, cells highly expressed p53 protein and upregulated its target genes, which triggered cell-cycle arrest, senescence and cell death by apoptosis or ferroptosis 90. The point-mutated codon 273 (G>A) of p53 pre-mRNA promoted its splicing through methylation of METTL3, leading to the over production of p53 R273H mutant protein that contributed to multidrug resistance in CRC 91.

### non-coding RNAs regulate m^6^A modification in CRC

**Figure 3:**
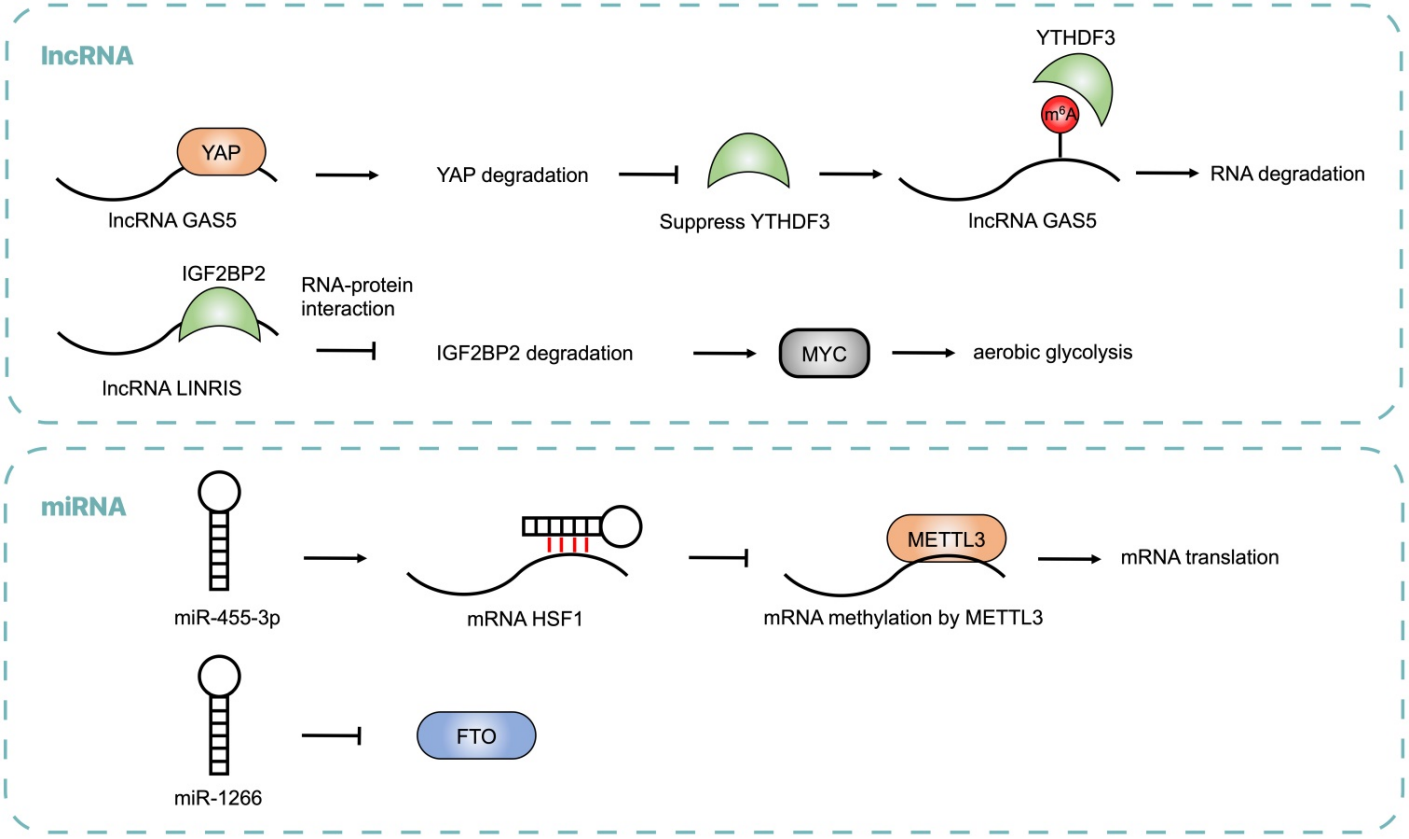
Noncoding RNAs, including lncRNA and miRNA, regulate m6A modification by modulating the expressions of m^6^A regulators, which is critical for CRC progression.

Emerging evidence showed that non-coding RNAs may regulate m^6^A modification by modulating the expression of m^6^A regulators and influence post-transcriptional gene expression in CRC (**Table 2 and Figure 3**). lncRNA LINRIS was upregulated in CRC patients with poor prognosis. LINRIS blocked K139 ubiquitination of IGF2BP2 and prevented its degradation through the autophagy-lysosome pathway (ALP), which promoted the MYC-mediated aerobic glycolysis in CRC cells 92. The transcription of LINRIS was inhibited by GATA3 in CRC cells, thus suppressing the proliferation of tumors both in orthotopic models and PDX models. Another lncRNA GAS5 could upregulate YTHDF3 through a YAP-dependent manner, while YTHDF3 could also recognize m^6^A of GAS5 to promote its decay, which formed a negative function loop of GAS5-YAP-YTHDF3 that contributed to CRC progression 93. Consistent with lncRNA, abnormal expression of miRNA was found in CRC. miR455-3p bound to 3'-UTR of HSF1 mRNA to block its interaction with METTL3 and repress its translation, thus inhibiting CRC progression. However, β-catenin suppressed the generation of miR455-3p and enhanced the expression of HSF1, which promoted glutaminolysis and activated mTOR in CRC 94. It was reported that miR-1266 was lowly expressed in CRC tissues and negatively regulated the expression of FTO, leading to the proliferation of CRC 95. The expression of miRNA-1266 was correlated to tumor size and TNM of CRC patients.

It has been found that non-coding RNAs are critical for cancer progression, but little is known about its function through an m^6^A-dependent manner. These results advance our understanding of non-coding RNA in cancer epigenetics.

## m^6^A as clinical determinants in CRC

### m^6^A as biomarkers of CRC

Most m^6^A regulators are dysregulated in CRC and their expression level was found correlated with clinical outcome of CRC patients, indicating the potential to become biomarkers for CRC 96. For instance, the downregulation of METTL14 is closely related to malignant progression and poor recurrence-free survival and overall survival of patients, suggesting the potential role of METTL14 in predicting tumor metastasis and recurrence 45. The expression of YTHDC2 was found to be positively correlated with the colon tumor stage, including metastasis 82. CRC patients with high expression of RBRP have a poor prognosis 86.

However, the same m^6^A regulator may exert oncogenic or antitumor function according to different studies. Some studies identified METTL3 as an oncogene associated with poor prognosis 37,39-42,91, while another study suggested that positive expression of METTL3 is correlated with longer survival time 46. Since the heterogeneity of m^6^A regulators made it difficult to detect cancer or predict prognosis, their target RNAs may be better biomarkers. Among various RNAs, circRNAs that can be detected in the blood are promising biomarkers. The m^6^A-modified circNSUN2 was found upregulated in serum and metastatic liver tissues of patients and was positively associated with CRC cell invasion, thus providing a novel diagnostic and prognostic predictor for colorectal liver metastasis 87. Future studies should examine whether the serum concentration of m^6^A regulators and their target RNAs is correlated to CRC diagnosis or prognosis, rather than their expression level in tumor tissues.

Due to the rapid development of bioinformatics, many software tools have been developed to predict cancers. Bioinformatic tool RNAMethyPro, a novel gene expression signature that comprised of seven m^6^A regulators, is used to predict prognosis in multiple cancers 97. Using comprehensive pan-cancer analysis, activated epithelial-mesenchymal transition (EMT) is identified as a highly conserved biological process across multiple cancer types, and further investigation on CRC revealed that high-risk patients were associated with the mesenchymal subtype, activated stromal infiltration and poor anti-EGFR therapeutic response 97.

### m^6^A as therapeutic target of CRC

Developing inhibitors of oncogenic m^6^A regulator like METTL3 and agonist of antitumor m^6^A regulator like METTL14 is a promising therapeutic strategy to overcome cancer, improve immune responses and reduce drug resistance.

Although it remains unknown how FTO participates in CRC tumorigenesis, FTO inhibitors have been widely explored as anticancer drugs in other types of cancer. Meclofenamic acid (MA) is one of selective FTO inhibitors by competing with FTO binding sites 98. Another inhibitor of FTO called FB23-2 has been developed to impair the proliferation and enhance differentiation of AML cells 99. R-2-hydroxyglutarate (R-2HG) inhibits FTO demethylase activity and elevates m^6^A level in leukemia cells by enhancing the degradation of MYC and CEBPA, thus displaying anti-tumor activity 100. Although some inhibitors have been applied to some types of cancer, there is still no application of these therapeutics in CRC patients.

The m^6^A modifications can regulate immune responses to anti‐PD‐1 therapy. As mentioned before, METTL3 or METTL14 KO as well as ALKBH5 KO in mice enhanced the efficacy of anti-PD-1 therapy 51,81. Using ALKBH5-specific inhibitor could lead to the similar phenotype, indicating the future translational application 81. Therefore, the inhibitors of m^6^A regulators can also be developed as an adjuvant therapy.

Mutations in m^6^A sites can induce multidrug resistance in CRC cells, so targeting m^6^A by regulating expression of m^6^A regulator is emerging as new therapeutics. For instance, mutant p53 proteins that promote drug resistance is a prospective therapeutic target. Either silencing METTL3 expression by using small interfering RNA or inhibiting RNA methylation with neplanocin A suppressed m^6^A formation in p53 pre-mRNA, and substantially increased its phosphorylation level which reduced p53 function in cells heterozygously carrying the R273H mutation, thereby re-sensitizing these cells to anticancer drugs 91.

Although targeting m^6^A seems a promising therapeutic strategy in addition to present treatments, the side-effect should not be ignored. RNA modification exists in all types of cells and exerts many fundamental function to maintain physiological function. Therefore, the application of inhibitors or agonist of m^6^A regulators may disturb its function in healthy organisms and lead to severe outcomes.

### Potential of Gut microbiota to alter m^6^A in CRC

Gut microbiota has been an area of intense focus of biological and clinical research 101. Gut microbiota comprises trillions of bacteria, viruses, fungi, archaea and protozoa, with their genome encoding numerous proteins that human cannot produce, which may have critical functions in physiological and pathological process 102. Dysbiosis of gut microbiota may lead to abnormal composition of microorganisms and impact human health. Increasing diseases are being demonstrated relevant to host microbiota, including inflammatory bowel disease 103, diabetes 104, cardiovascular diseases 105, neurological diseases 106, and various types of cancer 107,108. It was reported that gut microbiota might affect m^6^A deposition in the cecum and liver and influence pathways related to metabolism, inflammation and antimicrobial responses, which contributed to CRC tumorigenesis and metastasis 109. For instance, compared with conventional mice, METTL16 was downregulated in germ-free mice with its target mRNA Mat2a less methylated 109. Therefore, m^6^A modification represents a novel mechanism of interaction between host and commensal bacteria, setting the ground for future studies and promising therapies.

Increasing metabolites produced by gut microbiota have been demonstrated to influence CRC. Butyrate is a kind of short chain fatty acids as well as a classical intestinal microbial metabolite, which could modulate gut microbiota composition by increasing *Firmicutes* and *Proteobacteria*, and improve host immune response in mice with CRC liver metastasis 110. Butyrate inhibited CRC development through an m^6^A dependent manner by downregulating METTL3 and related cyclin E1 39. Recently, aspirin has been discovered to reduce the risk of CRC in human and this function was confirmed in mice that gut microbes could affect the bioavailability and protective effects of aspirin to prevent colon tumor formation 111. Further studies may examine whether oral administration of bacteria products or their metabolites could prevent cancer in human, minimizing the side effect of aspirin and maximizing its protective effect. Drugs other than aspirin can be tested to explore whether they exert the antitumor or oncogenic function through gut microbiota. Although accumulating evidence has been uncovering the relationship between gut bacteria and CRC tumorigenesis, there is little study on other types of microorganism, such as virus and fungi. Future studies should focus more on these species that colonize in intestinal tract which may also influence human health 112.

## Conclusions and perspectives

The incidence and mortality for CRC has been largely reduced by regular screening with fecal occult blood test and colonoscopy, starting at age 50 years 113. Patients diagnosed CRC at early stage benefit greatly from surgery and have a long overall survival. However, in the past few decades, there is an increasing trend of CRC patients diagnosed before age 50 years, also called early-onset CRC, with more evident for rectal cancer than colon cancer 114. Coincidentally, the expression patterns of m^6^A regulators are different in colon cancer and rectal cancer, indicating different m^6^A features between colon cancer and rectal cancer 96. Further, the overall survival of CRC patients at late stage is far from satisfactory, especially with liver metastasis. Therefore, the burden of CRC remains high in many countries.

The exploration of epigenetics is uncovering a new layer of cancer biology. As the most prevalent RNA modification in eukaryote, m^6^A has been detected in different types of RNAs to regulate post-transcriptional gene expression and participate in various biological processes. The m^6^A modification is raising increasingly broad concern due to cumulative evidence demonstrating that aberrant m^6^A level in RNAs may influence the occurrence and development of various types of cancers 115. For example, m^6^A modification in gastric cancer promoted EMT and metastasis through METTL3/ZMYM1/E-cadherin signaling 116. METTL3 promoted hepatocellular carcinoma progression through YTHDF2-dependent degradation of SOCS2 mRNA 117. FTO, highly regulated in certain AML subtypes, abolished m^6^A of ASB2 and RARA mRNA, which enhanced leukemogenesis and inhibited all-trans-retinoic acid (ATRA)-induced AML cell differentiation 76. Interestingly, it was reported that METTL3 was modified by small ubiquitin-like modifier (SUMO) in hepatocellular carcinoma, which promoted cancer progression through mediating Snail mRNA homeostasis 118. Since dysregulated m^6^A modification is found in various types of cancer, systematic analysis of molecular features and clinical relevance of m^6^A regulators may further improve our understanding of cancer biology 119.

In addition to m^6^A, other RNA modifications have also been reported in CRC, such as m^7^G and Nm. The maturation of miRNA let-7e, mediated by m^7^G methylation of METTL1, interfered the translation of high mobility group AT-hook 2 (HMGA2) mRNA, which inhibited the progression of colon cancer 120. lncRNA ZFAS1 stabilized multiple small nucleolar RNAs(snoRNA) and promoted Nm modification of rRNAs, thus regulating the RNA stability and translation of their downstream targets, leading to CRC initiation and maintenance 121. The coordination of different RNA modifications, such as m^6^A and m^5^C, has been reported 62. Moreover, the epigenetic modifications are not not isolated phenomena, but a complex regulatory network where multiple crosstalk of dysregulated modifications exists, which forms a comprehensive biological system 122,123.

There are two controversial issues on current researches of m^6^A.

Although both METTL3 and METTL14 are the core components of methyltransferase complex, METTL3 was upregulated while METTL14 was downregulated in CRC, which exerts oncogenic and antitumor effect respectively. The differences is partly because METTL3 is the catalytic unit while METTL14 is essential for stabilizing METTL3 conformation, substrates RNA binding and m^6^A sites deciding 124. Constructing mouse model of METTL3 knockdown and knockout as well as METTL14 knockdown and knockout may help explain their different role in cancer. Interactions between methyltransferase complex and its regulatory factors may also contribute to their biological function, which requires further elucidation.

The other one is the contradiction that MELLT3, the m^6^A writer that increases the RNA m^6^A level, is an oncogene in CRC, while FTO or ALKBH5, the m^6^A eraser that decreases the RNA m^6^A level, is also an oncogene in CRC. We made a hypothesis that if the writer installs m^6^A at a specific site of a certain RNA, and the eraser uninstalls m^6^A in the same site of the same RNA, they may regulate the RNA conversely and represent opposite roles in cancer: one is oncogenic and the other is antitumor. It was reported that depletions of METTL3 and ALKBH5 resulted in substantially decreased and increased expression of a subset of small GTPase mRNAs and proteins 125, which supported our hypothesis to some extent. Therefore, the targets, including the RNAs, m^6^A sites and downstream pathways, of writers and erasers that reported in different studies are different, which reveals different mechanisms in cancer carcinogenesis. However, only a modest number of small GTPases were modulated by METTL3 and ALKBH5 125, indicating other regulatory factors other than the writer and eraser. The m^6^A readers are crucial m^6^A regulators to exert post-transcriptional functions, which may account for the seemingly contradictory roles between the writer and eraser. Although some m^6^A methylases and demethylases have been identified, there are still potential m^6^A regulators undiscovered, which may participate in the regulation of m^6^A modification. Future studies could examine the effect of m^6^A writer and eraser on the same RNA simultaneously to explore whether there are other regulatory factors involved in m^6^A modification.

In conclusion, we summarized the current advances of m^6^A modification and its regulators in CRC, uncovering a novel dimension of cancer biology. Given that the m^6^A modification is one of numerous epigenetic modifications, it is essential to explore whether the m^6^A-mediated post-transcriptional regulation contributes more to the differences between the transcriptome and proteome. Despite the dual role of m^6^A regulators that exerted either oncogenic or antitumor role in various types of cancer, their downstream genes may become better biomarkers or therapeutic targets. The inhibitors of m^6^A regulators showed the potential of an adjuvant therapy to increase immune responses to anti-PD-1 therapy or reduce multidrug resistance. So far, there is no clinical application of therapeutic strategy targeting m^6^A in CRC. Therefore, more efforts should be made to elucidate the mechanism and develop novel treatments.

## Figures and Tables

**Table 1:** Roles of m^6^A Regulators in CRC

m^6^A Regulator	Role in CRC	Expression	m^6^A RNA	Mechanism	Ref.
Writer					
METTL3	oncogenic	↑	mRNA SOX2	enhance mRNA stability	40
	oncogenic	↑	mRNA HK2 and SLC2A1	enhance mRNA stability	41
	oncogenic	↑	mRNA CCNE1	enhance mRNA stability	39
	oncogenic	↑	pre-mRNA p53 (273G>A)	promote pre-mRNA splicing	91
	oncogenic	↑	miR-1246	promote pri-miRNA maturation	42
	oncogenic		mRNA Stat1 and Irf1	promote mRNA degradation	51
	oncogenic	↑			37
	antitumor	↓		modulate p38/ERK pathways	46
METTL14	antitumor	↓	lncRNA XIST	promote RNA degradation	43
	antitumor	↓	miR-375	promote pri-miRNA maturation	45
	antitumor	↓			37
	antitumor	↓	mRNA SOX4	promote mRNA degradation	44
	oncogenic		mRNA Stat1 and Irf1	promote mRNA degradation	51
WTAP	oncogenic	↓		target the WTAP-WT1-TBL1 axis to suppress Wnt/β-catenin signalling	55
	oncogenic	↑			37
RBM15	oncogenic	↑			37
ZC3H13	oncogenic	↑			37
KIAA1429	oncogenic	↑			37
ZCCHC4	oncogenic	↑			37
Readers					
YTHDC1	oncogenic	↑	circNSUN2	increase cytoplastic export	87
	oncogenic	↑			37
YTHDC2	oncogenic	↑		promote translation of HIF-1α	82
YTHDF1	oncogenic	↑		transcriptionally upregulated by c-Myc	84
	oncogenic	↑		activate Wnt/β-catenin signaling pathway	83
	oncogenic	↓			37
YTHDF3	oncogenic	↑	lncRNA GAS5	facilitate RNA degradation	93
IGF2BP2	oncogenic	↑	mRNA HMGA2	enhance mRNA stability	87
	oncogenic	↑	mRNA SOX2	enhance mRNA stability	40
HNRNPC	oncogenic	↑			37
RBRP	oncogenic	↑	mRNA c-Myc	enhance mRNA stability	86
Erasers					
FTO	oncogenic	↑			37
ALKBH5	oncogenic	↓	mRNA Mct4/Slc16a3	promote pre-mRNA splicing and enhance mRNA stability	81
	oncogenic	↓			37

**Table 2:** Roles of ncRNAs in CRC

RNA type	Role in CRC	m^6^A enzyme	Mechanism	Ref
**miRNA**				
miR-1266	antitumor	FTO	negatively regulate FTO	95
miR455-3p	antitumor	METTL3	bind to the target mRNA of METTL3 to block their interaction and repress its translation	94
**lncRNA**				
GAS5	antitumor	YTHDF3	suppress YTHDF3 through YAP-mediated pathway by facilitating YAP translocation, phosphorylation and degradation	93
LINRIS	oncogenic	IGF2BP2	prevent the degradation of IGF2BP2 from autophagy-lysosome pathway	92

## Abbreviations

ALKBH5: alkB homolog 5
ALP: autophagy-lysosome pathway
AML: acute myeloid leukemia
ANKLE1: Ankyrin Repeat and LEM Domain Containing 1
ATRA: all-trans-retinoic acid
CCNE1: Cyclin E1
CDS: coding sequence
circRNA: circular RNA
CpG: cytosine-phosphate-guanine
CRC: colorectal cancer
DART-seq: deamination adjacent to RNA modification targets
EMT: epithelial-mesenchymal transition
Flacc: Fl(2)d-associated complex component
FTO: Fat mass and obesity-associated protein
Gab2: GRB2 associated binding protein 2
GATA3: GATA binding protein 3
GLUT1: glucose transporter 1
HIF-1α: hypoxia-inducible factor 1α
HK2: hexokinase 2
HMGA2: high mobility group AT-hook 2
HNRNPA2B1: heterogeneous nuclear ribonucleoprotein A2B1
I: inosine
IFN: interferon
IGF2BPs: insulin-like growth factor 2 mRNA-binding proteins
IRF1: interferon regulatory factor 1
KO: knockout
lncRNA: long noncoding RNA
m^1^A: N^1^-methyladenosine
m^5^C: 5-methylcytidine
m^6^A-REF-seq: m^6^A-sensitive RNA-endoribonuclease-facilitated sequencing
m^6^A-SEAL: FTO-assisted m^6^A selective chemical labeling
m^6^A: N^6^ Methyladenosine
m^6^Am: N^6^,2′-O-dimethyladenosine
m^7^G: N^7^-methylguanidine
MA: meclofenamic acid
Mct4: monocarboxylate transporter 4
MeRIP-seq: methylated RNA immunoprecipitation sequencing
METTL14: methyltransferase like 14
METTL16: methyltransferase like 16
METTL3: methyltransferase like 3
miCLIP: m^6^A individual-nucleotide-resolution cross-linking and immunoprecipitation
miRNA: micro RNA
mRNA: message RNA
MSI-H: microsatellite instability-high
mt-tRNA: mitochondrial tRNA
mTOR: mammalian target of rapamycin
Nm: 2ʹ-O-methylation
NSUN2: NOL1/NOP2/SUN domain family member 2
PAR2: proteinase-activated receptor 2
PD1: programmed cell death protein 1
PDX: patient-derived xenograft
PI3K: phosphatidylinositol-3-kinase
pri-miRNAs: primary miRNAs
R-2HG: R-2-hydroxyglutarate
RBM15/15B: RNA-binding motif protein 15/15B
RBRP: RNA-binding regulatory peptide
SLC16A3: solute carrier family 16 member 3
snoRNA: small nucleolar RNAs
SNPs: single-nucleotide polymorphisms
snRNA: small nuclear RNA (U6)
SOX: SRY (sex determining region Y)-box
SPRED2: Sprouty related EVH1 domain containing 2
STAT1: signal transducer and activator of transcription 1
TBL1: transducin β-like protein 1
tRNA: transfer RNA
UTR: untranslated region
vtRNA: vault RNA
WTAP: Wilms' tumor 1-associating protein
XIST: X-inactive specific transcript
YAP1: Yes-associated protein 1
YTH: YT521-B homology
YTHDC1-2: YTH domain-containing protein 1-2
YTHDF1-3: YTH domain family protein 1-3
ZC3H13: zinc finger CCCH domain-containing protein 13
Zeb1: zinc finger E-Box binding homeobox 1
Ψ: pseudouridine
